# Complication pattern of chronic suppurative otitis media in North Indian subjects

**DOI:** 10.6026/973206300222134

**Published:** 2026-04-30

**Authors:** Rajeev Kumar Nishad, Priyanka Das, Reetu Verma

**Affiliations:** 1 Department of ENT, Autonomous State Medical College, Firozabad, Uttar Pradesh, India; 2 Department of ENT, Himalaya Medical College & Hospital, Patna, Bihar, India; 3 Department of Anaesthesiology, Autonomous State Medical College, Firozabad, Uttar Pradesh, India

**Keywords:** Chronic suppurative otitis media (CSOM), mastoiditis, meningitis, subdural abscess, complications, otitis media

## Abstract

Chronic suppurative otitis media (CSOM) remains a significant otologic condition in India, with possible intracranial and extracranial complications despite advances in therapy. Hence, this retrospective study evaluated 7,940 confirmed CSOM cases, identifying 92 (1.2%) with complications 69.6% intracranial and 47.8% extracranial, with 16 subjects presenting both types and a male-to-female ratio of 2:1. The 21-30 year age group showed the highest involvement (32.6%), with meningitis (32.6%) and subdural abscess (15.2%) as the leading intracranial and mastoiditis (37%) as the most common extracranial complication. Although improved antibiotic and surgical interventions have reduced overall incidence, mortality and morbidity remain elevated due to delayed diagnosis and intervention. Hence, we report the contemporary burden and patterns of CSOM related complications in the Indian population and emphasizing timely management to prevent severe outcomes.

## Background:

The occurrence of CSOM (chronic suppurative otitis media) and its associated complications has shown a significant reduction with the use of newer, more effective antibiotics. However, in developing nations such as India, these infections pose a major healthcare burden and challenges concerning the diagnosis and management of CSOM. Also, CSOM is a major and threatening disease owing to the complications associated with the disease [1]. The use of antibiotics in CSOM has played a significant role in managing CSOM and the associated otitis complications, which have become increasingly infrequent; however, these complications are still seen and early identification of clinical patterns linked to these complications has resulted in more efficacious treatment [2]. Complications of chronic ear diseases, such as CSOM, usually present during acute exacerbations of infection. These exacerbations create additional therapeutic challenges, as effective management of the underlying disease is essential to prevent recurrence and associated complications [3]. In cases where the infection progresses to intracranial complications, disease spread may occur through various routes, including direct extension from the mastoid cavity or middle ear, or via hematogenous dissemination. A lack of familiarity among newer otologists with otogenic intracranial complications (IC) can lead to delayed diagnosis and management [4]. Therefore, it is of interest to assess the pattern, incidence and mortality of complications associated with CSOM (chronic suppurative otitis media), which is a major health challenge in India.

## Materials and Methods:

The current retrospective study was conducted to evaluate the mortality, incidence and pattern of complications related to chronic suppurative otitis media (CSOM), a significant health issue in India. The study was conducted at the Department of ENT, F.H. Medical College, Etmadpur, Agra, Uttar Pradesh. Before participation, each subject provided both verbal and written informed consent. During the specified study period, the study evaluated 7940 participants diagnosed with CSOM who presented with concerns following CSOM at the Institute. All subjects who did not provide informed consent for study participation were removed, as were those with missing data and medical records and those who did not show up for follow-up. The information gathered for research included age, investigations carried out, gender, symptoms of concern, treatment provided, complications encountered and treatment results and was obtained from the study participants' medical records following their participation. The study then separated 92 participants who experienced difficulties linked to CSOM. Subjects with CSOM issues were further classified into two main groups: IC (intracranial complications) and EC (extracranial complications). Lateral sinus thrombosis, subdural abscess, brain abscess and/or meningitis were examples of intracranial problems. Petrositis, facial nerve paralysis, labyrinthitis and mastoiditis were the extracranial consequences. In the majority of the study subjects, CT (computed tomography) scans of the petro-mastoid and brain were done and records were available. In most subjects, appropriate and necessary steroids and antibiotics were given. Subjects who had meningitis were managed using steroids and antibiotics. Physicians regularly performed aural toileting and ENT surgeons administered antibiotics during dressing. Subjects that had intracranial abscesses underwent surgery as burr hole aspiration with craniotomy or burr hole aspiration without craniotomy. Mastoid surgeries were done in subjects with extracranial complications. In two subjects, simultaneous neurosurgical and ontological interventions were done. Statistical analysis of the gathered information was carried out using SPSS (Statistical Package for the Social Sciences) software version 24.0 (IBM Corp., Armonk, NY, USA) to evaluate descriptive measures, Student's t-test, ANOVA (analysis of variance), Mann-Whitney U test and Chi- square test. The Pearson correlation coefficient was used to assess correlations among different parameters. The results for the work were presented as the mean and standard deviation, along with the frequency and percentage expressions. The p-value considered was <0.05.

## Results:

The present retrospective study was conducted to evaluate the mortality, incidence and pattern of complications related to chronic suppurative otitis media (CSOM), a significant health issue in India. A total of 7,940 participants diagnosed with CSOM and presenting with related concerns were included. Complications were observed in 1.2% of these individuals (n = 92). Among them, 69.6% (n = 64) experienced intracranial complications, while 47.8% (n = 44) had extracranial complications. Multiple complications occurred in 16 of the 92 individuals. The male-to-female ratio among study participants was 2:1. Regarding age distribution, 32.6% (n = 30) of participants were in the 21-30-year age group, 30.4% (n = 28) were aged 11-20 years, 21.7% (n = 20) were aged 1-10 years, 8.7% (n = 8) were aged 41-50 years and 6.5% (n = 6) were in the 31-40-year range (Table 1 - see PDF). The most frequently reported intracranial complication was meningitis (32.6%, n = 30), followed by subdural abscess (15.2%, n = 14). Among extracranial complications, mastoiditis was the most common, reported in 37% (n = 34) of cases (Table 1 - see PDF). Based on clinical symptoms, otorrhea was present in all participants (100%, n = 92), followed by fever (91.3%, n = 84), convulsions (69.6%, n = 64), loss of consciousness (65.2%, n = 60), headache or severe headache (63%, n = 58), otalgia (43.5%, n = 40), hearing loss (41.3%, n = 38), post-aural swelling (30.4%, n = 28), facial paralysis (10.9%, n = 10) and vertigo (2.2%, n = 2) (Table 2 - see PDF). Mortality due to intracranial complications occurred in 28.1% (n = 18) of cases. Management of meningitis in all 30 patients included systemic antibiotics and corticosteroids, along with daily topical antibiotic dressings and aural toileting. Burr hole aspiration was performed in 19.6% (n = 18) of cases with intracranial complications, while craniotomy and drainage were performed in 13% (n = 12). For extracranial complications, cortical mastoidectomy and incision and drainage were performed in 17.4% (n = 16) and 13% (n = 12) of cases, respectively.

## Discussion:

The present retrospective study assessed 7940 subjects with a confirmed CSOM diagnosis and reported complications after CSOM at the Institute within the considered time frame. Among 7940 subjects managed at the Institute with CSOM, 1.2% (n=92) had complications. Intracranial and extracranial complications were seen in 69.6% (n=64) and 47.8% (n=44) study subjects, respectively. In 16 of 92 subjects, more than one complication was seen. Recent tertiary-center cohorts report complication rates of 1-10%, with extracranial predominance (52-80%) over intracranial events (11-46%), contrasting with our higher intracranial burden, likely reflecting late referrals [5]. The male-to-female ratio in the study participants was 2:1. These data shared similarity with the data from work by Verhoeff *et al.* in (2006) [6] and Adoga *et al.* in (2014) [7], where subjects considered by the authors depicted the demographic data comparable to the present study in subjects with CSOM. This male predominance and demographic profile align with contemporary Indian and global series [8]. On considering the age distribution in the study subjects, it was highlighted that majority of personnel in the study had the age in category of 21-30 years contributed by 32.6% (n=30) subjects with second common being 30.4% (n=28) subjects from age contribution of 11-20 years, 21.7% (n=20) subjects in the age range of 1-10 years, 8.70% (n=8) subjects in the age range of 41-50 years and least 6.50% (n=6) subjects in the age range of 31-40 years. A recent comprehensive review confirms CSOM complications disproportionately affect young adults and children in low-resource settings, mirroring our age distribution [4]. The most common intracranial complication was meningitis, seen in 32.6% (n=30) personnel and the second most common was subdural abscess in 15.2% (n=14) subjects. In contrast, the most common extracranial complication was mastoiditis in 37% (n=34) of study subjects, respectively. These results were consistent with the findings of Monasta *et al.* in (2012) [9] and Mostafa *et al.* in (2009) [10], in which the age distribution and the distribution of CSOM complications mentioned in these works were consistent with the present work. Indian series consistently describe mastoiditis and meningitis/subdural abscess as dominant extracranial and intracranial manifestations, validating our patterns [11]. According to the study's findings, most common clinical symptom was contributed by otorrhea occurring in 100% (n=92) of the subjects, with fever being the second most common in 91.3% (n=84), convulsions were noted in 69.6% (n=64), loss of consciousness was depicted by 65.2% (n=60), headache was noted in 63% (n=58), otalgia was encountered in 43.5% (n=40), hearing loss was reported by in 41.3% (n=38), post-aural swelling was reported by 30.4% (n=28), paralysis of facial structure was evident in 10.9% (n=10) and vertigo was seen in 2.2% (n=2) of the study subjects. These results were consistent with the work of Dubey and Larawin (2007) [12] and Ologe and Nwawolo (2003) [13], who reported clinical symptoms identical to those observed in the current work.

Contemporary data reinforce otorrhea (universal), fever, convulsions and headache as key red flags for CSOM complications, emphasizing early recognition [14]. 28.1% (n=18) of the study participants had intracranial mortality. This exceeds mortality in recent series with prompt surgery (0-5%) and highlights the risks of delayed intervention [5]. Antibiotics and steroids were used for management in all 30 patients with meningitis, along with daily topical antibiotic dressings and aural toileting. Burr hole aspirations were performed in 19.6% (n=18) of the participants with intracranial problems, while craniotomy and drainage were performed in 13% (n=12) of the subjects. Cortical mastoidectomy and incision and drainage were performed on 17.4% (n=16) and 13% (n=12) of the participants, respectively, for extracranial problems. These findings were consistent with those of Trimis *et al.* (2003) [15] and Kong *et al.* (2009) [16], whose reports of CSOM complications and deaths were consistent with the present results. Ongoing reviews note persistent uncertainty regarding optimal antibiotics, supporting our multimodal approach (antibiotics and surgery) [2]. Recent evidence indicates rising complicated CSOM cases despite antibiotics, driven by access gaps. Our large Indian cohort advances knowledge by quantifying contemporary intracranial dominance (69.6%), age burden (21-30 years peak) and 28.1% mortality, urging referral pathways and early imaging [17]. Another study by Borah *et al.* (2024) [18] on bacterial infections in chronic suppurative otitis media (CSOM) cases at a tertiary care center in North-East India reports similar findings, with Pseudomonas aeruginosa (27.7%) and Staphylococcus aureus (including MRSA in 17%) as predominant pathogens, mirroring regional microbial patterns in CSOM complications.

## Conclusions:

Data shows that CSOM complications have decreased due to more effective and modern antibiotics and active surgical illness care. However, mortality and morbidity rates are still elevated and CSOM problems are still considered common among Indian participants. An increase in disease-related death and morbidities can be attributed to delays in treatment for intracranial concerns and diagnosis. Further longitudinal research with larger sample sizes and longer monitoring periods is necessary in the future.
